# Comprehensive characterization of immune- and inflammation-associated biomarkers based on multi-omics integration in kidney renal clear cell carcinoma

**DOI:** 10.1186/s12967-019-1927-y

**Published:** 2019-05-27

**Authors:** Enyang Zhao, Lihong Li, Wenfu Zhang, Wanhui Wang, Yunhui Chan, Bosen You, Xuedong Li

**Affiliations:** Department of Urinary Surgery, The Second Affiliated Hospital of Harbin Medical University, Harbin, 150006 Heilongjiang People’s Republic of China

**Keywords:** Immune, Inflammation, Kidney renal clear cell carcinoma, Network modules, Multi-omics molecular, DNA methylation

## Abstract

**Background:**

Kidney renal clear cell carcinoma (KIRC) is the most common type of kidney cancer in adults, and it is responsible for approximately 90–95% of cases. Although extensive evidence has suggested that many immune- and inflammation-related genes could serve as effective biomarkers in KIRC, the potential associations among immune-, inflammation- and KIRC-related genes has not been sufficiently understood.

**Methods:**

Here, we integrated multiple levels of data to construct an immune-, inflammation- or KIRC-directed neighbour network (IIKDN network) and a KIRC-related gene-directed network (KIRCD network).

**Results:**

Our analysis suggested that immune- and inflammation-related genes in the network have special topological characteristics and expression patterns related to KIRC. We further identified five core clusters that showed a tighter network structure and stronger correlation of expression from the KIRCD network. Specifically, multiple-level molecular characteristics were systematically portrayed, including somatic mutation, copy-number variant and DNA methylation for the genes in five core clusters. We discovered that the genes showed strong correlation with respect to the expression and methylation levels in these five core clusters. These five core clusters could become special prognostic biomarkers for KIRC, and functional analysis showed that they were associated with activation of the immune and inflammation systems and cancer progression.

**Conclusions:**

Our findings highlighted the novel role of the immune and inflammation genes in KIRC.

**Electronic supplementary material:**

The online version of this article (10.1186/s12967-019-1927-y) contains supplementary material, which is available to authorized users.

## Background

Renal cell carcinoma (RCC) is a type of kidney cancer and is the leading cause of cancer-related death, with an annual incidence of more than 270,000 new cases globally [1]. RCC is the most common type of kidney cancer in adults and is responsible for approximately 90–95% of cases. Clear cell renal cell carcinoma (ccRCC), also known as kidney renal clear cell carcinoma (KIRC), is the most common (~ 80%) subtype [2]. Genetic changes including alterations in genes that control cellular oxygen sensing and the maintenance of chromatin states will induce underlying KIRC [3]. In recent years, there has been significant progress in the survival of KIRC patients as a result of the evolution of the frontline treatment paradigm for KIRC, particularly for intermediate-risk and poor-risk patients [4]. However, the effectiveness and safety of the current treatment strategy should be further discussed and improved. Thus, there is a need to develop strategies that combine immunotherapy and molecular biomarkers as novel treatments in the clinic.

In the development, growth, and progression of cancer, the tumour microenvironment is essential and important [5]. Immune disorder and chronic inflammation are two classic and prevalent examples of ongoing perturbation within the microenvironment [6]. In addition, evading immune destruction and tumour-promoting inflammation are two essential hallmarks of cancer [7]. In recent years, studies have uncovered the basic principles and process of inflammation and inflammatory signalling that promote cancer and the response of cancer to therapy [8]. Chevrier et al. constructed an immune atlas of KIRC and revealed potential biomarkers and targets for immunotherapy development in KIRC [9]. Chang et al. used a systemic inflammation score to predict postoperative prognosis of patients with KIRC [10]. Inflammation is a fundamental innate immune response to perturbed tissue homeostasis [11]. The relevance among the immune response, inflammation and KIRC is complex and important. Characterizing the mechanistic relationship between KIRC and its inflammation and immune microenvironment could lead to the identification of novel biomarkers or therapeutic targets for KIRC treatment. Although KIRC is closely related to the immune response and inflammation, the global characteristics of the immune response and inflammation in KIRC are unknown.

The Cancer Genome Atlas (TCGA) is a comprehensive and coordinated effort to accelerate our understanding of the molecular basis of cancer, and it has generated comprehensive, multi-dimensional maps of the key genomic changes in 33 types of cancer. The comprehensive molecular characterization for KIRC has been depicted [12]. Sato et al. have integrated multiple molecular analysis and uncovered novel associations among DNA methylation, gene mutation and/or gene expression and copy-number profiles, enabling the stratification of clinical risks for KIRC patients [13]. These findings demonstrate that integration of multiple omics data could help in evaluating the mechanism and developing the therapy of KIRC. However, the integrated molecular analysis regarding immune and inflammation genes in KIRC is insufficient.

In this study, we constructed an immune-, inflammation- or KIRC-directed neighbour network (IIKDN network) and a KIRC-related gene-directed network (KIRCD network) to address the role of the immune response and inflammation in KIRC. We also analysed the topological features and expression pattern of the IIKDN and KIRCD networks. We identified five modules from the KIRCD network, and the genes in these five modules were mostly differentially expressed and indicated that the five modules play important roles in KIRC. In addition, we discovered some hub modules related to the immune response and inflammation that were strongly co-expressed in these five modules. These modules all included several key immune-related, inflammation-related and KIRC-related genes, which demonstrated the functional significance of the immune- and inflammation-related genes to KIRC. Specially, we systematically portrayed multiple level molecular characteristics including somatic mutation, copy-number variant and DNA methylation for the genes in the five modules. These modules could serve as prognostic biomarkers for KIRC, and functional analysis showed that they were associated with activation of the immune system and cancer process. Our findings highlighted the novel role of the immune and inflammation genes in KIRC. These comprehensive analyses can serve as important resources for future experimental dissection of biomarkers in KIRC.

## Methods

### Human immune and inflammation-related gene datasets

All the genes related to the immune response and inflammation from AmiGO 2 version: 2.4.26 in Homo sapiens species were downloaded [14]. Finally, we collected 3068 immune-related genes from 651 records and 604 inflammation-related genes from 91 records.

### KIRC-related gene datasets

DisGeNET is the largest publicly available database that collects genes and variants associated with human diseases [15]. Finally, we extracted 585 KIRC-related genes.

### Human protein–protein interaction data

We downloaded protein–protein interaction (PPI) data from the Human Protein Reference Database (HPRD) database and constructed the human protein interaction network [16].

### Construction of network and analysis of topological characteristics

First, we constructed a sub-network of IIKDN network from the PPI network, and the IIKDN network included immune-, inflammation-, and KIRC-related genes and their direct interacting genes in the network (referred to as neighbour genes). Next, we extracted all KIRC-related genes from the IIKDN network to construct a KIRC-related gene-directed network (KIRCD network). Finally, the Cytoscape software was used to construct the network and analyse the topological properties of nodes in both IIKDN and KIRCD networks [17].

### Identification of core clusters from KIRCD network

We used the MCODE tool of Cytoscape, following the default parameters, to identify all the network modules from the KIRCD network (http://apps.cytoscape.org/apps/mcode). MCODE could cluster a network follow topology to obtain densely connected regions. We identified several clusters and extracted five clusters with the highest MCODE scores.

### Characteristics of gene expression pattern of KIRCD network and core clusters

The gene expression profile of KIRC was obtained from The Cancer Genome Atlas (TCGA) (https://cancergenome.nih.gov/). The KIRC gene expression profile contains 536 KIRC patients and 72 matched normal samples. Pearson correlation coefficients (PCCs) values between two nodes, either in the KIRCD network or core clusters, were calculated via the gene expression data of KIRC patients. The relationship was considered as significantly co-expressed if the p values and false discovery rate (FDR) values were lower than 0.05 and 0.1. We also identified differentially expressed genes between the KIRC and matched control samples via the unpaired t-test for all the genes in the five core clusters (P < 0.05, FDR < 0.1).

### Integrated multiple level molecular analysis of genes in core clusters

Somatic mutation, copy-number variant (CNV), and DNA methylation data on KIRC were acquired from the TCGA Pan-Cancer project. GENCODE (Release 28, GRCh38) annotation files, including comprehensive gene annotations in a GTF format, were used for mapping the somatic mutations, CNVs and DNA methylation site-specific genes. We extracted the score < − 0.2 as copy-number deletion and > 0.2 as copy-number amplification. We also used the unpaired t-test to identify differential DNA methylation sites. PCC values were calculated to estimate the co-expression pattern of the most significant differential DNA methylation sites for each gene.

### Survival analysis for the core clusters in KIRC

We used the regression coefficient of each gene in the core cluster related to patient survival based on gene expression data to verify if these core clusters were associated with survival. First, the KIRC samples were randomly divided into two groups and the samples in these two groups are independent. Next, a multivariate Cox regression model was used for each gene in the cluster to obtain a standardized Cox regression coefficient for the first group. Age, cancer stage and sex also become confounders in this process. We established a risk score formula for each KIRC patient based on the expression values of each selected gene for the held-out group weighed by their estimated regression coefficients, following the above multivariate Cox regression analysis. In other words, to avoid the overfitting, the risk scores were constructed by holding back a part of the KIRC dataset during the Cox regression analysis and using the held-out samples to validate the model. Second, we used the median of the risk score as the threshold value to divide the KIRC patients into high-risk and low-risk groups. Finally, Kaplan–Meier survival analysis was performed for the high- and low-risk groups, and statistical significance was assessed using the log-rank test. The survival results were considered significant when P < 0.05 and FDR < 0.1. All analyses were performed within the R 3.3.3 framework.

### Functional enrichment analysis for the core clusters

With the Enrichr tool online web server using default parameters, functional enrichment was performed for genes across core clusters [18]. We obtained enriched GO terms (P < 0.05, FDR < 0.1), KEGG pathways (P < 0.05, FDR < 0. 1).

## Results

### Immune and inflammation-related genes play crucial roles in KIRC

**Fig. 1:**
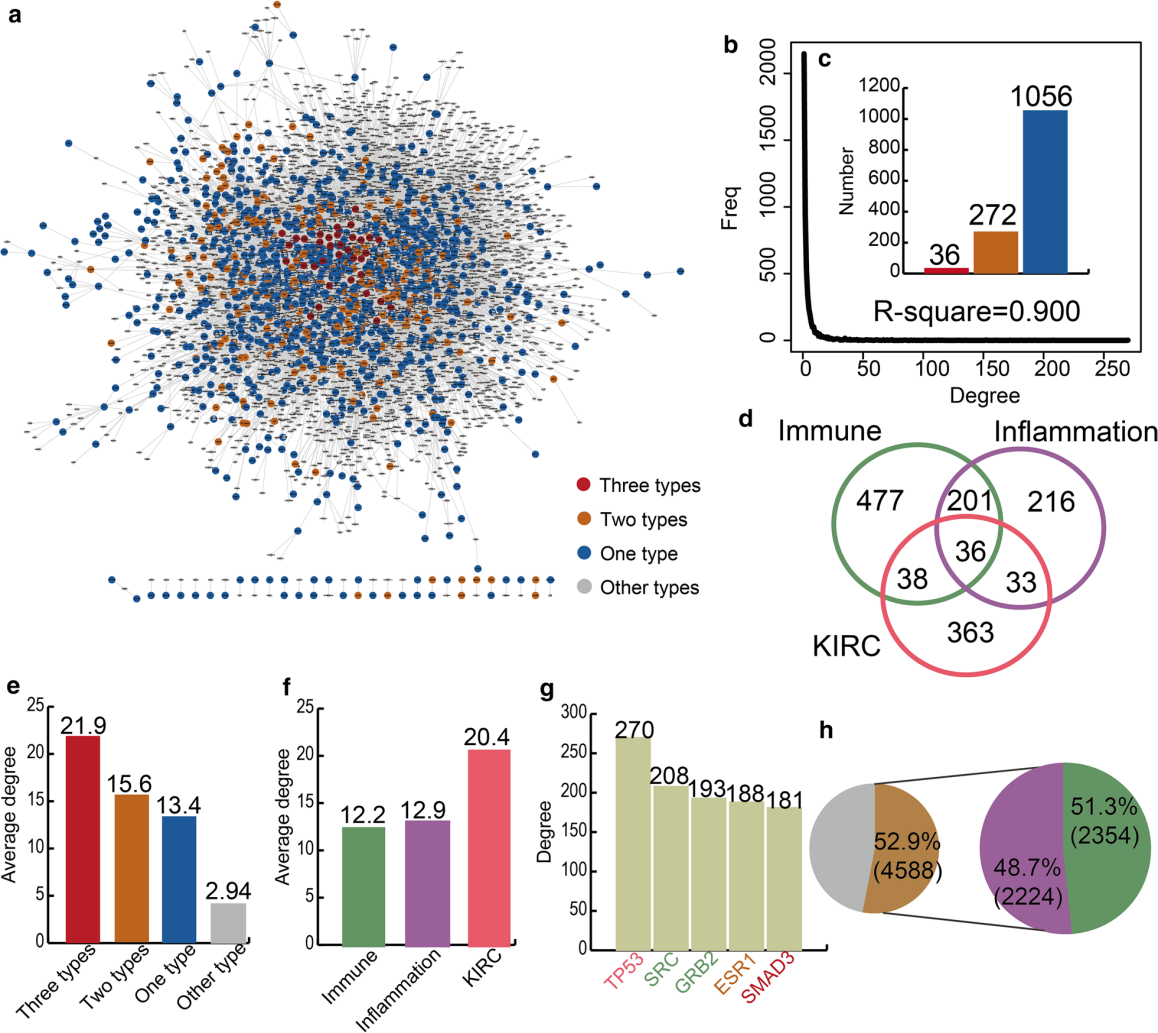
The properties of immune, inflammation or KIRC-directed neighbour network (IIKDN network). **a** The global IIKDN network. **b** The degree distribution of nodes. **c** The bar plot shows the number of diverse genes. **d** The Venn diagram shows the number of immune-, inflammation- and KIRC-related genes. **e** The bar plot shows the average degree of diverse kinds of genes. **f** The bar plot showed the average degree of immune-, inflammation- and KIRC-related genes. **g** The top five genes ranked by gene degrees including TP53, SRC, GRB2, ESR1 and SMAD3. The bar plot shows the degree of the five genes. **h** The pie chart shows the percent of immune- and inflammation-related genes associated with KIRC genes

We constructed an immune, inflammation or KIRC-directed neighbour network (IIKDN network), which is a sub-network of the PPI network (Fig. 1a). The IIKDN network contained 5391 nodes and 15,411 edges. The degree of all the nodes in the IIKDN network showed a scale-free distribution (R-square = 0.900) (Fig. 1b). We also divided the genes in the IIKDN network into four types. We defined the gene as “three types” if the gene was immune-related, inflammation-related gene and KIRC-related. We defined the gene as “two types” if the gene was both immune- and inflammation-related or immune- and KIRC-related or inflammation- and KIRC-related. We defined the gene as “one type” if the gene was only immune-related or inflammation-related or KIRC-related. We defined the gene as “other type” if the gene belonged to none of the above mentioned gene types. In the IIKDN network, there were 36 “three types” genes, 272 “two types” genes and 1056 “one type” gene (Fig. 1c, d). We found that the “three types” genes had the highest degree (average degree = 21.9), and the average degree of the “other type” genes was far lower than that for the immune-, inflammation- and KIRC-related genes (Fig. 1e). The result suggested immune- and inflammation-related genes play more essential roles than other genes in KIRC. The result also indicated complex links among the immune-related, inflammation-related and KIRC-related genes. We also discovered that the average degree of KIRC-related genes was the highest and showed that KIRC-related genes still play essential roles in the IIKDN network (Fig. 1f). The top five genes including TP53, SRC, GRB2, ESR1 and SMAD3 exhibit a direct connection and the highest degree in the network (Fig. 1g). Notably, SRC and GRB2 were immune-related genes. SMAD3 was a “three type” gene, and ESR1 was an inflammation-related and KIRC-related gene, which indicated that immune- and inflammation-related genes play hub roles in the IIKDN network. More than half of the interactions of KIRC-related genes were associated with immune- and inflammation-related genes (Fig. 1h). The number of other gene neighbours was smaller than that of immune- and inflammation-related gene neighbours for KIRC-related genes. All the above results indicated that immune- and inflammation-related genes play crucial roles in KIRC.

### Immune- and inflammation-related genes directly interact with KIRC-related genes and identification of core clusters

**Fig. 2:**
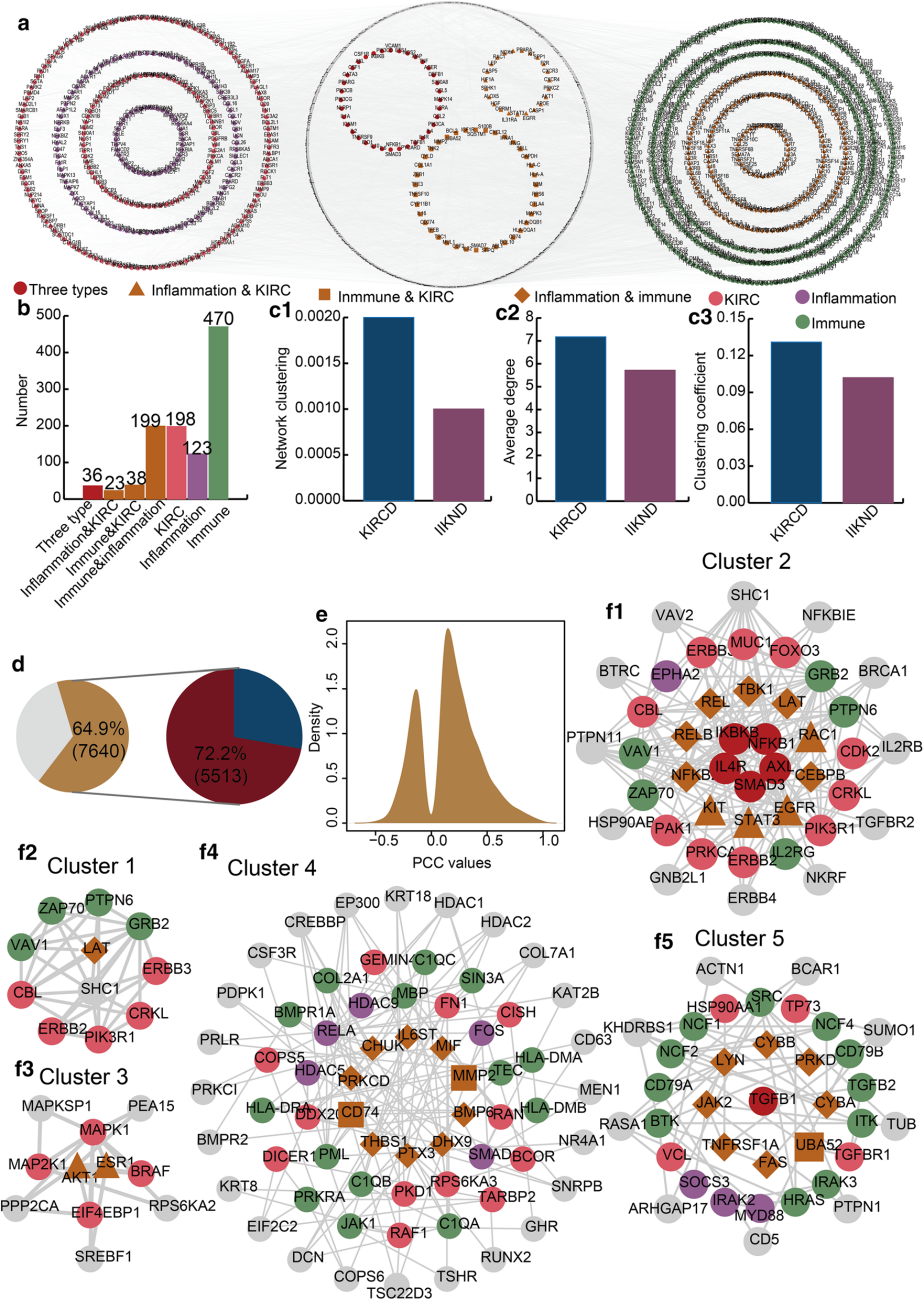
The characteristics of KIRC-related gene-directed network (KIRCD network) and identification of core clusters. **a** The global KIRCD network. **b** The number of diverse genes. **c1**–**c3**) The bar plots show the comparison of topological characteristics between KIRCD and IIKND. **d** The first pie chart shows the percent of significantly correlated interactions. The second pie chart shows the percent of positive and negative correlated interactions. **e** The density distribution curve of PCC values in the KIRCD network. **f1**–**f5**) The five core modules identified from the KIRCD network

We extracted a KIRC-related gene-directed network (KIRCD network) from the IIKDN network to further explore the association among immune-related, inflammation-related and KIRC-related genes (Fig. 2a). The KIRCD network only contained the KIRC-related genes and genes directly connected to them. There were 3360 nodes and 12,058 edges in the KIRCD network. We found 853 immune- and inflammation-related genes in the KIRCD network (Fig. 2b). Specially, we discovered that the nodes in the KIRCD network exhibited a higher network clustering, average degree and clustering coefficient than the nodes in the IIKDN network (Fig. 2c1–c3). This result of comparing topological features indicated that the KIRCD network is closer in structure.

Next, we further explored the associations among immune-, inflammation- and KIRC-related genes on the expression level based on the gene expression profile of KIRC patients. In the KIRCD network, there were 64.9% significant co-expressed interactions and 72.2% positive co-expressed interactions (Fig. 2d). We observed that the density curves for the PCC values showed classical bimodal distribution (Fig. 2e). The above results that we analysed in the KIRCD network indicated that the association among immune-related, inflammation-related and KIRC-related genes not only showed on the topological structure but also showed on the expression pattern. Next, we revealed the communications among immune-related, inflammation-related and KIRC-related genes by performing a module analysis. We extracted five core clusters with top scores (Fig. 2f1–f5). We discovered that the immune-, inflammation- and KIRC-related genes interacted in each cluster. These core clusters demonstrated the close relationships among the immune response, inflammation and KIRC.

### Characteristics of expression pattern for core clusters to reveal the associations among immune-, inflammation- and KIRC-related genes

**Fig. 3:**
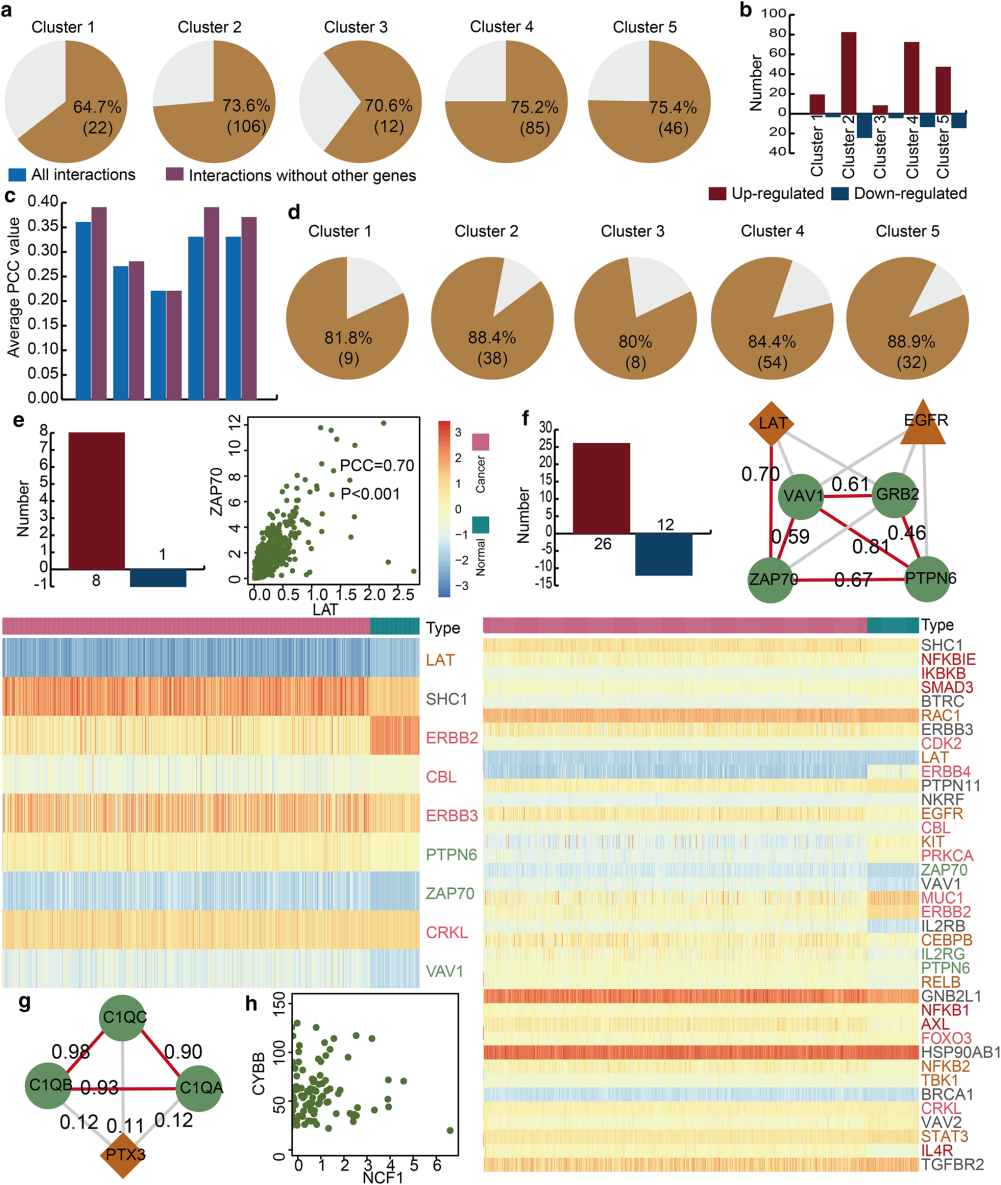
The expression patterns of five core clusters. **a** The pie charts show the percent of differentially expressed genes. **b** The bar plot shows the number of up- and down-regulated genes. **c** The bar plot shows the average PCC values of all interactions and interactions without other genes. **d** The pie charts show the percent of significantly correlated interactions. **e**–**h** The core clusters. The bar plots show the number of up-regulated genes and down-regulated genes for each core cluster. The point plots show the expression pattern between two significant gene interactions. The heat maps show the differential expression between KIRC patients and matched normal samples for all differentially expressed genes

The core clusters showed close structure features, and we further considered if they also hold close communications on the expression level. First, we characterized the co-expression pattern of all genes in five core clusters. Most interactions in all five core clusters were significant co-expressed, and they indicated that most genes showed strong correlations (Fig. 3a). We also discovered that immune-, inflammation- and KIRC-related genes had higher PCC values when other genes were removed in the five clusters (Fig. 3b). We also identified differentially expressed genes in each core cluster based on the expression profile of KIRC and control samples after describing the co-expression pattern. We discovered that more than 80% genes in the core clusters showed significant differential expressions (Fig. 3c). In all five core clusters, there were more up-regulated genes than down-regulated genes (Fig. 3d). We further analysed the core clusters and found some strongly correlated gene pairs and functional modules. For example, in the first core cluster, there were 8 up-regulated and 1 down-regulated genes, and 81.8% genes were differentially expressed (Fig. 3e). The immune- and inflammation-related gene LAT and the immune-related gene ZAP70 showed significant differential expression in KIRC patients (P = 4.79e−55, 4.08e−65). LAT and ZAP70 showed strong positive correlation (PCC = 0.7, P < 0.001) and indicated that some immune- and inflammation-related genes play their roles by interacting in KIRC. In the second core cluster, most genes were differentially expressed (Fig. 3f). We also found a functional module including four immune-related genes, an inflammation-related and KIRC-related gene EGFR and an inflammation-related and immune-related gene LAT (Fig. 3f). Most of these six genes showed strong positive correlation, and this close interacting functional module may play an important role in KIRC. A similar phenomenon was observed in the fourth and fifth core clusters (Fig. 3g, h). In the fourth core cluster, three immune-related genes encoded complement C1q subcomponent subunit family genes including C1QA, C1QB and C1QC. The three genes all showed strong positive correlation (PCC = 0.98, 0.90 and 0.93) and formed a functional immune-related module in KIRC. All the above results revealed that the immune-, inflammation- and KIRC-related genes play their roles in KIRC by interacting within these five core clusters.

### Core clusters showed complex genomic characteristics including somatic mutation, CNV and DNA methylation

**Fig. 4:**
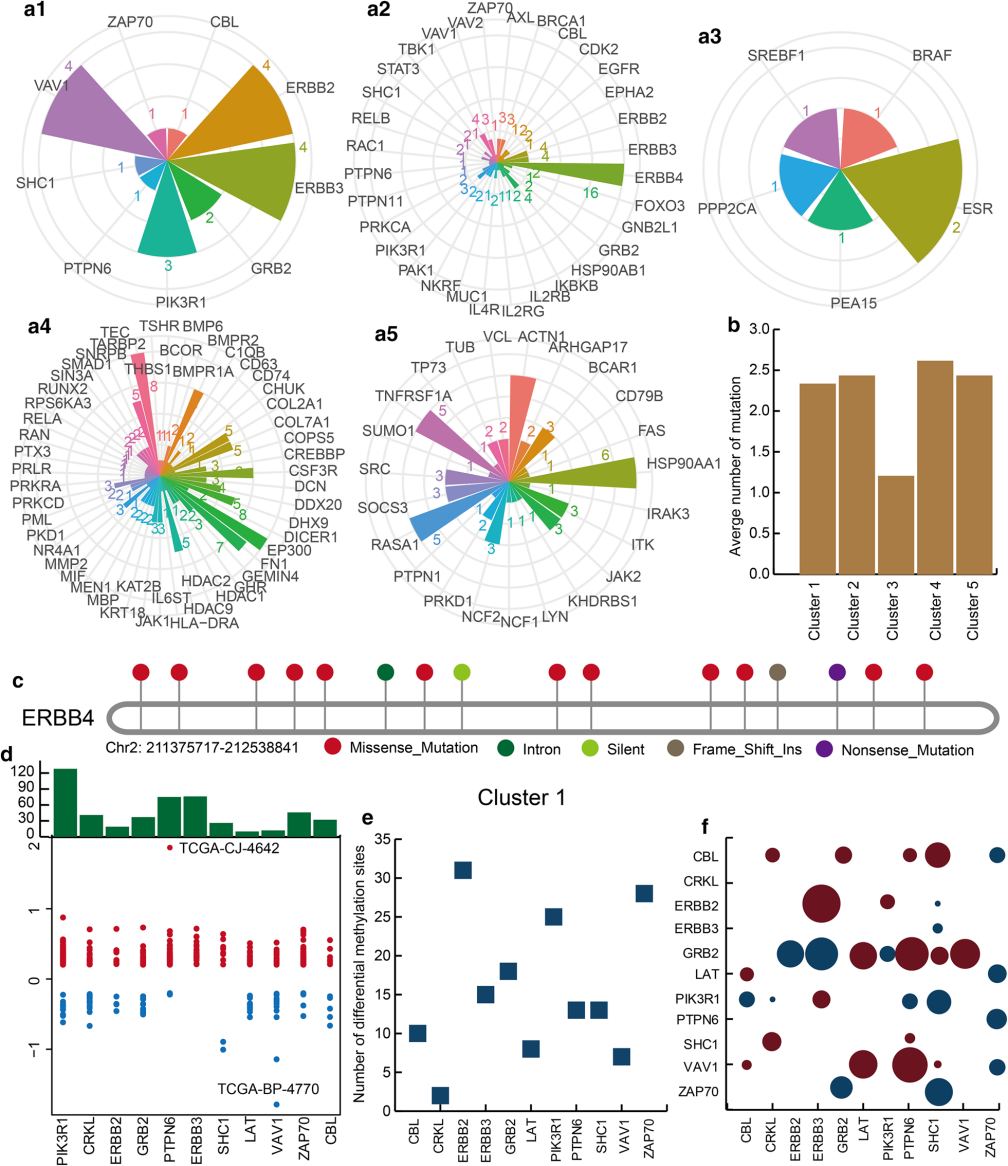
Complex genomic characteristics including somatic mutation, CNV and DNA methylation. **a1**–**a5** The rose diagrams show the number of somatic mutations for each gene in the five core clusters. **b** The bar plot shows the average number of mutations in the five core clusters. **c** An example, ERBB4, is shown. **d** The CNV pattern in the first core cluster. Red and blue represent amplification and deletion. **e** The point plot shows the number of differential methylation sites in the first core cluster. **f** The co-methylation pattern of each core cluster; the larger circle represents stronger correlation, red represents positive correlation, and blue represents negative correlation

Multi-dimensional genomic analysis promoted the depth of understanding for the associations among immune-, inflammation- and KIRC-related genes in core clusters. First, we discovered that most genes contained a certain number of somatic mutations in all core clusters (Fig. 4a1–a5). However, the numbers of somatic mutation in different genes were diverse. In addition, we found that the average number of somatic mutations in all clusters were almost similar (Fig. 4b). In all core clusters, the gene ERBB4 contained the highest number of somatic mutations, and other genes in the second cluster contained less somatic mutations. The mutation types on ERBB4 were complex, including missense mutation, intron mutation, silent mutation, frame shift insert and nonsense mutation (Fig. 4c). Specifically, ERBB4 interacted with the KIRC-related genes ERBB2, ERBB3 and MUC1 and the immune-related gene GRB2. Next, we discovered that certain genes contained CNV in the core clusters. For example, all genes in the first core cluster contained CNVs including amplification and deletion (Fig. 4d). PIK3R1 was a famous KIRC-related gene, and more than 120 samples exhibited CNV alterations in this gene. Other immune-related genes such as PTPN6 also contained some CNV alterations. Through somatic mutation and CNV analysis, we found that cancer genes usually contained more genomic alterations than immune- and inflammation-related genes. It could be inferred that immune- and inflammation-related genes could play a synergistic role with KIRC-related genes in KIRC.

Next, we also analysed the DNA methylation pattern for the genes in core clusters and found that most genes exhibited differential DNA methylation. For example, all genes in the first cluster had differential DNA methylation sites (Fig. 4e). Similar to somatic mutation and CNV, the KIRC-related gene ERBB2 exhibited the most differential DNA methylation sites. Notably, we discovered that the genes showed strong correlations with respect to the methylation level, similar to gene expression, and this indicated the interactions between immune, inflammation and KIRC-related genes and DNA methylation level (Fig. 4f). The network structure, expression pattern and DNA methylation pattern showed coincident correlation among immune-, inflammation- and KIRC-related genes in core clusters.

### Core clusters in KIRC has prognostic potential

**Fig. 5:**
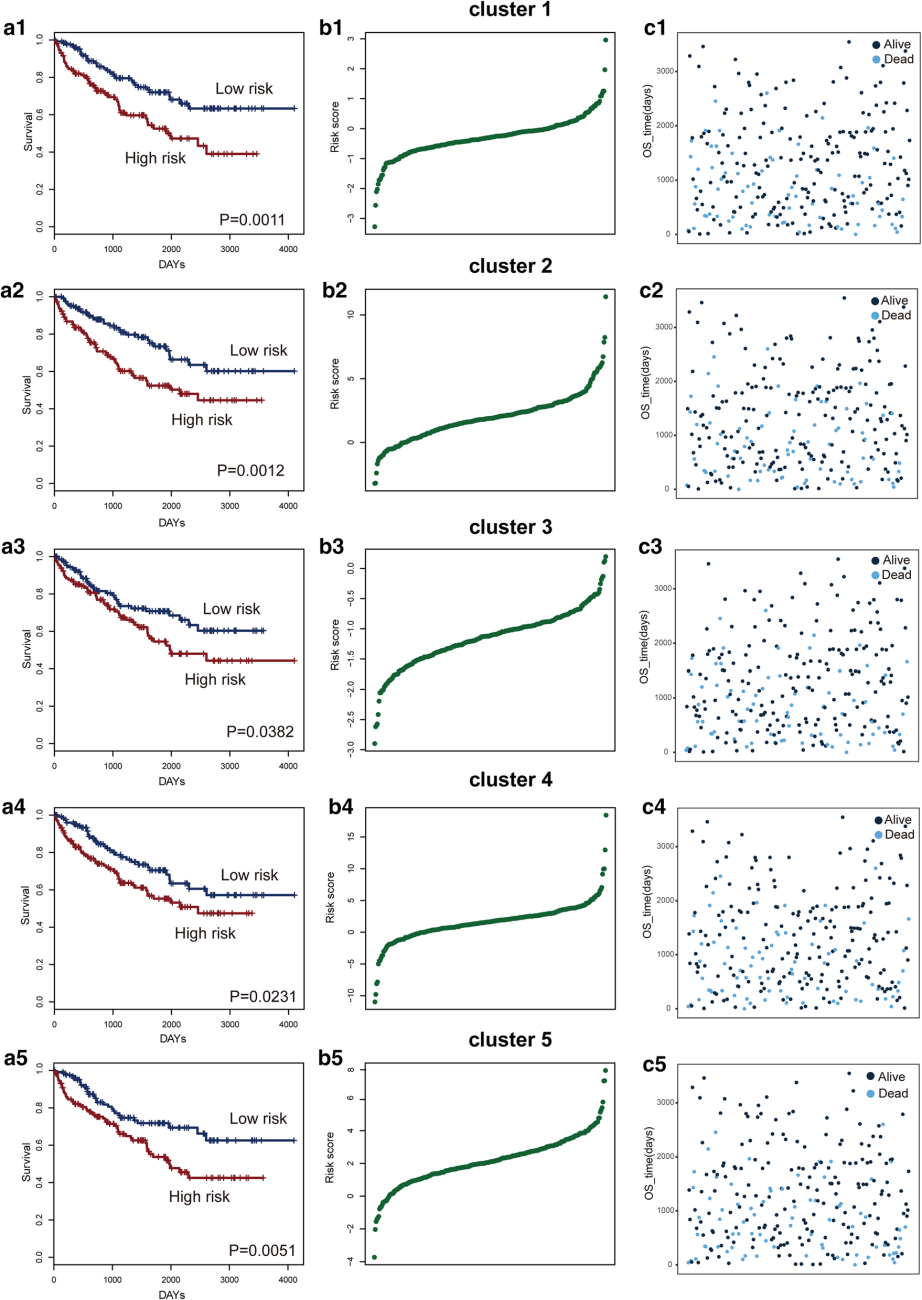
Core clusters are associated with survival for KIRC patients. **a1**–**a5** The Kaplan–Meier curve for the overall survival of two patient groups with high- and low-risk scores in the KIRC patient set. The difference between the two curves was evaluated by a two-sided log-rank test. **b1**–**b5** The gene-based risk score distribution of the genes in each cluster. **c1**–**c5** The patient survival status of the genes

To evaluate the potential value of core clusters as prognostic biomarkers in KIRC, we created a risk-score formula according to the expression of all the genes in each core cluster to generate OS (overall survival) prediction (see “Methods” section). We used median risk score as the cut-off point to test the survival of the KIRC patients. We calculated the risk scores of all the genes in each cluster for each patient and then ranked the patients according to their risk score. Next, the KIRC patients would be divided into high-risk or low-risk groups. All five core clusters were significantly associated with survival, and they could serve as prognostic biomarkers (Fig. 5a1–a5). In addition, KIRC patients in the high-risk group exhibited a significantly shorter median OS than those in the low-risk group. KIRC patients could be grouped based on the risk score of these genes in the core clusters (Fig. 5c1–c5, d1–d5). The results indicated that immune-, inflammation- and KIRC-related genes could collectively influence KIRC patient survival and serve as specific prognostic biomarkers.

### Core clusters associated with critical biological functions and the JAK/SAT signalling pathway

**Fig. 6:**
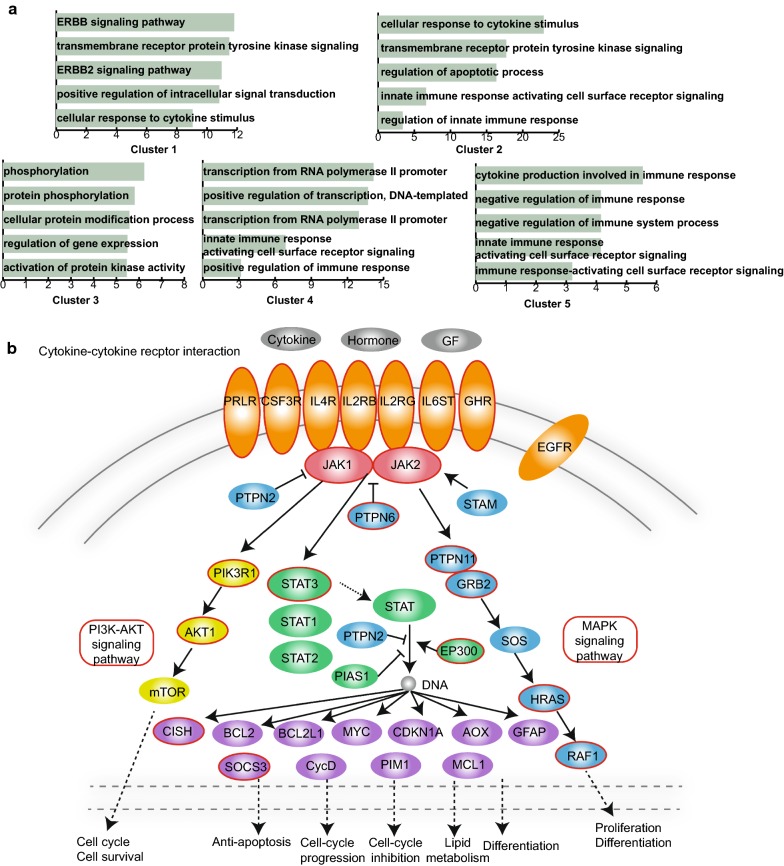
Functional analyses of five core clusters. **a** GO terms enriched for genes in the five core clusters, respectively, ranked by − log10(P) are presented as bar plots. **b** The KEGG pathway, the JAK/SAT signalling pathway and the genes in the core clusters are coloured red

We performed GO enrichment analysis based on all the genes in five core clusters, respectively. We found that these genes were enriched in different GO terms (Fig. 6a, Additional file 1: Table S1). We found that the genes in the second, fourth and fifth core clusters were associated with certain immune-related GO terms such as regulation of the innate immune response, innate immune response activating cell-surface receptor signalling and negative regulation of immune system system. In addition, some protein modification related GO terms including protein phosphorylation and activation of protein kinase activity were discovered. Studies have reported the important role played by aberrant phosphorylation in oncogenesis and immune disorders [19].

Notably, we discovered that the genes in these five core clusters were all related to the JAK/SAT signalling pathway (Fig. 5b). The JAK/SAT signalling pathway is now recognized as an evolutionarily conserved signalling pathway employed by diverse cytokines, interferons, growth factors, and related molecules [20]. The changes in the pathway are functionally relevant in various human diseases, especially cancer and immune-related conditions [21]. Not only have genome-wide association studies demonstrated that the JAK/SAT signalling pathway is highly related to human autoimmunity but also targeting JAKs is now a reality in immune-mediated disease. Cytokine–cytokine receptor interaction is an important part of this pathway, and these cytokines are essential for immune and inflammatory responses [22]. In our analysis, we found that several genes in the five core clusters play essential roles in this pathway, which indicated that these key genes identified by us were highly associated with the immune response and inflammation.

## Discussion

Our analyses provide novel insights into the study and treatment of KIRC by exploring the functional significance and molecular mechanism of immune and inflammation-related genes and following the interaction network, expression pattern, somatic mutation, CNV and DNA methylation data. Although some effective biomarkers in KIRC have been identified by previous studies, there has been no focus on global and system analysis of the roles of immune and inflammation-related genes in KIRC [23, 24]. In our analysis, we performed a network-based strategy to identify the KIRC-related PPI network and core clusters. The network-based strategy could not only consider the associations among immune-, inflammation- and KIRC-related genes but also provide global interactions between genes. The identification and characteristics of core clusters, which were identified from the network, provide novel and specific biomarkers for KIRC. Notably, the core clusters could serve as prognostic biomarkers and predict the survival for KIRC patients in the clinic. In addition, these core clusters were enriched in the JAK/STAR signalling pathway, and they provided novel insights regarding immunotherapy for KIRC.

We integrated multiple omics data including interaction network, expression, somatic mutation, CNV and DNA methylation data to globally depict core clusters. We found that the immune-, inflammation- and KIRC-related genes in the five core clusters were strongly correlated with respect to expression and methylation level. This result indicated that the consistency changed at multiple levels of the genome and transcriptome for the genes in core clusters. A similar phenomenon has been observed in other diseases [25, 26]. We also found that immune- and inflammation-related genes contained fewer somatic mutations compared with KIRC-related genes. We inferred that although immune- and inflammation-related genes could not directly cause KIRC, they played a role by cooperating with cancer oncogenes. Although the immune response and inflammation were related, the immune-related genes played more essential roles because they were higher in number in the five core clusters in KIRC. The functional analyses showed that there were distinctions and connections between the five core clusters. For example, we found that the genes in the second, fourth and fifth core clusters were associated with certain immune-related GO terms. However, other differences and detailed connections should be explored in future works. In addition, the GO term enrichment analysis could generate more accurate results by correcting for the background set of genes.

The induction and the maintenance of the chronic inflammatory response is a universal mechanism of immune tolerance [27]. Inflammatory factors induce accumulation of anti-inflammatory and immunosuppressive factors, resulting in a positive–negative feedback loop. In the present work, each core cluster contained immune-, inflammation- and KIRC-related genes, which indicated that these genes exhibited close interactions. We also found that many immune- and inflammation-related genes were strongly correlated with respect to expression and methylation in KIRC. We identified a key pathway, the JAK/SAT signalling pathway, which is based on genes in core clusters and which further demonstrated the roles of immune- and inflammation-related genes in KIRC. The immune- and inflammation-related genes were enriched in the upstream part of the JAK/SAT signalling pathway, which indicated that they play important roles in the early stage of KIRC. In addition, our method identified novel candidates associated with KIRC development and prognosis, which require further research and experimental validation.

## Conclusions

In summary, an immune, inflammation or KIRC-directed neighbour network (IIKDN network) and a KIRC-related gene-directed network (KIRCD network) were constructed to address the role of the immune response and inflammation in KIRC. Five core clusters were identified, and differential expression and co-expression pattern were depicted. In addition, multiple level molecular characteristics were systematically portrayed, including somatic mutation, copy-number variant and DNA methylation for the genes in the five modules. These five core clusters were associated with survival for KIRC patients, and functional analysis showed that they were associated with activation of the immune system and cancer process. Our findings provide novel insights into KIRC by considering the associations among inflammation, the immune response and KIRC.

## Additional file

**Additional file 1: Table S1.** The enrichment GO terms for Cluster 1.

## Data Availability

Not applicable.

## Abbreviations

KIRC: kidney renal clear cell carcinoma
RCC: renal cell carcinoma
ccRCC: clear cell renal cell carcinoma
TCGA: The Cancer Genome Atlas
GO: gene ontology
KEGG: Kyoto encyclopaedia of genes and genomes
IIKDN network: immune, inflammation or KIRC-directed neighbour network
KIRCD network: KIRC-related gene-directed network
PPI: protein–protein interaction
PCC: Pearson correlation coefficients
CNV: copy-number variant
